# Integrative multi-omics analysis reveals a membrane tension signature associated with tumor-stroma crosstalk in hepatocellular carcinoma

**DOI:** 10.3389/fimmu.2026.1978174

**Published:** 2026-10-05

**Authors:** Pengfei Zhu, Pengcheng Zhu, Zijuan Zhu, Gaozhong Bian, Yungang Liu, Qifan Yang, Peipei Luo

**Affiliations:** 1 Hebei Medical University, Shijiazhuang, China; 2 Hangzhou Dianzi University, Hangzhou, China; 3 The Second Affiliated Hospital of Bengbu Medical College, Bengbu, China; 4 Department of Oncology, Wujin Hospital Affiliated with Jiangsu University, Changzhou, China; 5 Department of Gastroenterology, Wujin Hospital Affiliated with Jiangsu University, Changzhou, China

**Keywords:** CFL1, hepatocellular carcinoma, MDK signaling, membrane tension, prognostic signature, tumor-stroma crosstalk

## Abstract

**Background:**

Dysregulated membrane tension (MT) drives tumor cell migration, invasion, and immune evasion, yet the prognostic value of MT-related genes (MTRGs) in hepatocellular carcinoma (HCC) remains unclear.

**Methods:**

We integrated transcriptomic and clinical data from TCGA, ICGC, and GEO. A three-gene MTRG prognostic signature was developed through univariate and LASSO-Cox regression, and validated using Kaplan–Meier analysis, time-dependent ROC curves, and calibration plots. Functional enrichment, immune infiltration, tumor mutational burden, and immunotherapy response was predicted using the Tumor Immune Dysfunction and Exclusion (TIDE) algorithm. Single-cell and spatial transcriptomics were used to investigate the mechanistic role of CFL1 in tumor-stroma crosstalk.

**Results:**

The signature (CFL1, CRTC2, SRGAP2) stratified HCC patients into high- and low-risk groups with distinct overall survival (OS). High-risk tumors showed coordinated activation of MT and ECM-remodeling pathways alongside an immunosuppressive microenvironment. In silico analyses suggested that CFL1-high tumor cells may transmit MDK signals to fibroblasts (via SDC2, NCL, and ITGA4–ITGB1) and to T cells (via NCL). Spatial transcriptomics confirmed predominant CFL1 enrichment in stromal compartments. The signature was an independent prognostic factor, and the nomogram showed promising clinical utility.

**Conclusions:**

This three-gene MTRG-associated signature is associated with predicted immunotherapy response based on computational analysis in HCC. Our results suggest a potential link between biophysical MT and tumor stroma co evolution and represent a potential therapeutic direction warranting further experimental validation.

## Introduction

Hepatocellular carcinoma (HCC) ranks as the sixth most common malignancy worldwide and the third leading cause of cancer-related death, with a 5-year OS rate of approximately 22% (1, 2). Although surgical resection, liver transplantation, targeted therapy, and immunotherapy have improved outcomes for selected patients, prognosis remains poor for many, reflecting the scarcity of reliable biomarkers and incomplete understanding of tumor biology (3, 4). Novel biomarkers and therapeutic targets are therefore required to refine patient stratification and enable personalized treatment (5–7). Beyond genetic and epigenetic alterations, the physical properties of the tumor microenvironment (TME)—including elevated solid stress, increased matrix stiffness, altered fluid shear stress, and tissue-level architectural remodeling—have emerged as critical regulators of malignancy (8–11). C Cells sense these mechanical cues and transduce them into biochemical signals through mechanotransduction pathways, thereby modulating tumor cell migration, invasion, and immune evasion (12–15). MT has attracted particular attention in this context: epithelial cells maintain higher MT than metastatic tumor cells, which constrains tumor dissemination by antagonizing BAR family protein activity (16). Conversely, reduced MT facilitates membrane protrusion and cell motility, promoting metastatic spread. Moreover, matrix stiffness, a major determinant of MT, induces CD8+ T cell exhaustion through the biomechanical checkpoint Osr2, thereby dampening immunotherapy efficacy (17). These findings underscore the pivotal role of membrane tension in tumor progression and immune regulation. Nevertheless, the molecular mediators that link MT to tumor aggressiveness and immune evasion remain largely undefined.

MTRGs constitute a functionally coherent set of molecules that regulate or respond to changes in MT, encompassing cytoskeletal regulators (e.g., CFL1), signaling transducers (e.g., CRTC2), and membrane-associated scaffolding proteins (e.g., SRGAP2). Although individual MTRGs have been implicated in cancer cell motility, their collective prognostic value and functional coordination within the HCC microenvironment have not been systematically characterized.

Here, we integrated multi-omics data from TCGA, ICGC, and GEO to construct and validate a three-gene MTRG prognostic signature (CFL1, CRTC2, SRGAP2). We further used single-cell and spatial transcriptomics to elucidate the mechanistic role of CFL1—the dominant contributor to the signature—in remodeling the TME. Our findings suggest that CFL1-high tumor cells may establish MDK-mediated communication with fibroblasts and T cells, potentially creating an MT-driven, stroma-reinforced immunosuppressive niche. This work provides a potentially clinically applicable prognostic tool and identifies a CFL1–MDK–stroma axis that links biophysical MT to tumor-stroma crosstalk and immune evasion in HCC.

## 2 Materials and methods

### 2.1 Data acquisition and preprocessing

**Figure 1** illustrates the overall study design. RNA expression, clinical and somatic mutation data of 371 HCC and 50 normal tissues were obtained from TCGA (https://portal.gdc.cancer.gov/), 240 samples from ICGC-LIHC (https://dcc.icgc.org/projects/LIRI-JP) were used as an independent validation set. HCC datasets (GSE121248, GSE45267) were retrieved from GEO for gene expression validation. A total of 84 MTRGs were curated from previous studies (18–23), encompassing genes involved in cytoskeletal regulation, membrane curvature sensing, mechanotransduction, and membrane trafficking. The complete gene list is provided in **Supplementary Table S1**.

**Figure 1:**
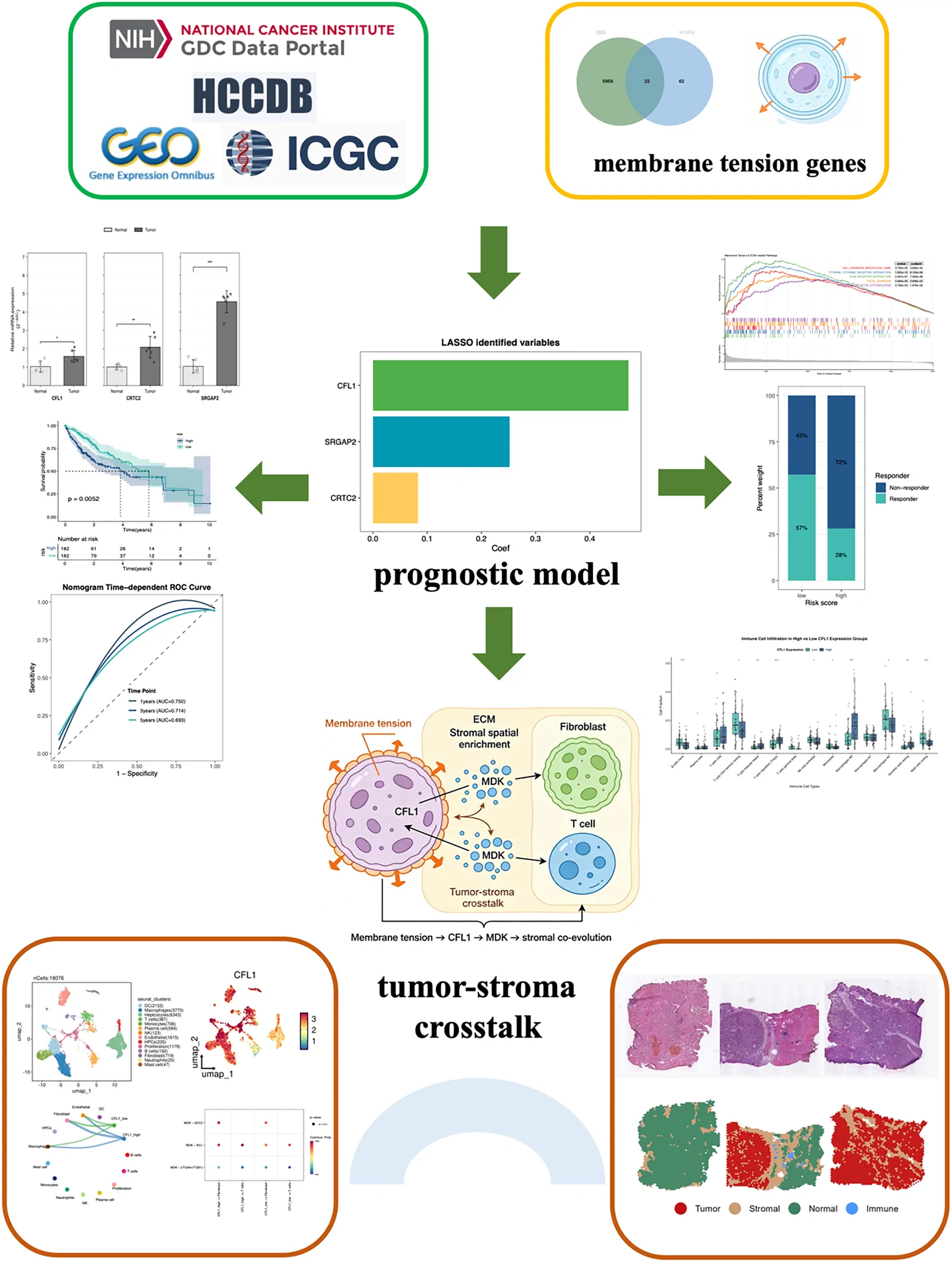
Graphical overview of the study design.

### 2.2 Construction and validation of the prognostic risk model and nomogram

Differentially expressed genes (DEGs) between HCC and adjacent normal tissues were identified using the Wilcoxon signed-rank test with |logFC| > 1 and FDR < 0.05. Intersection of DEGs with the 84 MTRGs yielded 22 candidate genes. Prognosis-related MTRGs were then selected by univariate Cox regression (R package “survival”), and a parsimonious three-gene signature was constructed using LASSO-Cox regression. The risk score was calculated for each patient using z-score normalized expression values as follows: Risk Score = (0.252 × SRGAP2) + (0.083 × CRTC2) + (0.471 × CFL1). Patients were stratified into low- and high-risk groups by the median risk score. The prognostic performance was evaluated by Kaplan–Meier survival analysis, time-dependent ROC curves (R package “timeROC”), and PCA in both the TCGA training cohort and the ICGC-LIHC external validation cohort. The independent prognostic value of the risk score was further assessed by univariate and multivariate Cox regression analyses incorporating age, gender, tumor stage, and T stage. To facilitate individualized survival prediction, a nomogram integrating the MTRG risk score and the above clinical parameters was constructed to estimate 1-, 3-, and 5-year OS probabilities. Nomogram performance was assessed by ROC curves, C-index, and calibration curves.

### 2.3 Functional enrichment, immune infiltration, and immunotherapy response prediction

To elucidate the biological pathways associated with the MTRG risk signature, Gene Set Enrichment Analysis (GSEA) was performed using the R packages “clusterProfiler”, “org.Hs.eg.db”, and “enrichplot” with the “c2.cp.kegg_legacy.v2023.2.Hs.symbols.gmt” gene set (FDR < 0.05). Given that CFL1 carries the highest coefficient weight (0.471) within the three-gene signature and represents the dominant prognostic contributor, we next sought to explore the association between CFL1 expression and the immune microenvironment. To this end, TCGA-LIHC patients were stratified into CFL1-high and CFL1-low groups based on median CFL1 expression, and immune cell fractions were quantified using CIBERSORT (R package “CIBERSORT”) with the LM22 signature matrix. TIDE algorithm was employed to predict immunotherapy response between high- and low-risk groups. Tumor mutational burden (TMB) was calculated as the total number of non-synonymous somatic mutations per megabase (Mb) using TCGA somatic mutation data processed by the “maftools” R package.

### 2.4 Single-cell and spatial transcriptomics analysis

Single-cell RNA sequencing (scRNA-seq) data of HCC were obtained from the GSE242489 dataset. Quality control filtering was applied to remove: (i) cells with fewer than 200 detected genes; (ii) cells with more than 6,000 detected genes (potential doublets); (iii) cells with mitochondrial gene proportion exceeding 20%; and (iv) genes detected in fewer than 3 cells. After filtering, a total of 41,246 cells (23,170 from normal liver and 18,076 from paired HCC tissues) were retained for downstream analysis. Data were normalized using the LogNormalize method, and the top 2,000 highly variable genes were selected. Principal component analysis (PCA) was performed, and the top 20 principal components were used for UMAP dimensional reduction and clustering (resolution = 0.5). Cell clusters were annotated based on canonical marker genes as follows: hepatocytes (ALB, APOA2), fibroblasts (COL1A2, ACTA2), endothelial cells (PECAM1, VWF), macrophages (C1QB、C1QC), T cells (CD3D, IL7R), NK cells (NKG7, GZMK), and B cells (CD79A, MS4A1). CellChat analysis was employed to infer cell-cell communication networks between CFL1^+^ and CFL1^−^ tumor cells and other cell populations, with particular focus on MDK-mediated signaling pathways. Spatial transcriptomics data were obtained from the HCCDB database (http://lifeome.net/database/hccdb2). Spots were annotated based on histopathological regions (tumor, stroma, normal, immune). The spatial expression patterns of CFL1 were visualized across different tissue regions, and average expression levels within each annotated region were calculated to validate the spatial distribution of model genes.

### 2.5 Preliminary qRT-PCR validation in clinical samples

To validate the expression of the three model genes, paired HCC and adjacent non-tumor tissues were collected from 6 patients who underwent surgical resection at Wujin People’s Hospital Affiliated to Jiangsu University. Total RNA was extracted using TRIzol reagent (Invitrogen, USA), and RNA integrity was assessed using an Agilent 2100 Bioanalyzer. Reverse transcription was performed using the FastKing RT Kit (QIAGEN, Germany) according to the manufacturer’s instructions. Quantitative real-time PCR was carried out on a LightCycler^®^ 480 System (Roche, Switzerland) using SYBR Green Premix (Takara, Japan). Relative gene expression was calculated using the 2^−△△CT^ method, with GAPDH as the internal control. Primer sequences are listed in **Supplementary Table S1**. In parallel, the expression of the three genes was also validated in independent GEO datasets (GSE45267 and GSE121248) using the “limma” R package.

### 2.6 Ethics statement

This study was approved by the Ethics Committee of Wujin People’s Hospital Affiliated to Jiangsu University (approval No. 2026-SR-044). Written informed consent was obtained from all participants prior to sample collection.

### 2.7 Statistical analyses

All statistical analyses were performed using R software (version 4.2.2). Univariate Cox regression and LASSO-Cox regression (10-fold cross-validation) with the “glmnet” package were used to construct the prognostic signature, with the penalty parameter λ selected by minimum partial likelihood deviance. Kaplan-Meier survival curves were compared using the log-rank test. Time-dependent ROC curves were generated using the “timeROC” package. Normality was assessed using the Shapiro-Wilk test; between-group comparisons were performed using the Wilcoxon test for non-normally distributed variables and Student’s t-test for normally distributed data. All reported P-values were two-sided, with Benjamini-Hochberg (BH) adjustment applied for multiple testing where appropriate. Statistical significance was defined as P < 0.05.

## 3 Results

### 3.1 Identification of a three-MTRG prognostic signature in HCC

Univariate Cox regression on 22 MTRGs in TCGA-LIHC identified nine prognosis-associated genes, among which CFL1 (HR = 1.970), CRTC2 (HR = 1.713), and SRGAP2 (HR = 1.622) showed the strongest hazard ratios (all P<0.001, **Figure 2A**). LASSO-Cox regression further condensed these into a parsimonious three-gene signature with the risk formula: Risk Score = (0.471×CFL1) + (0.083×CRTC2) + (0.252×SRGAP2), where CFL1 contributed the dominant weight (**Figure 2B**). Expression validation in two independent GEO datasets (GSE45267, GSE121248) and preliminary qRT-PCR validation in 6 paired clinical samples suggested upregulation of all three genes in tumors versus adjacent non-tumor tissues (**Figures 2C-E**).

**Figure 2:**
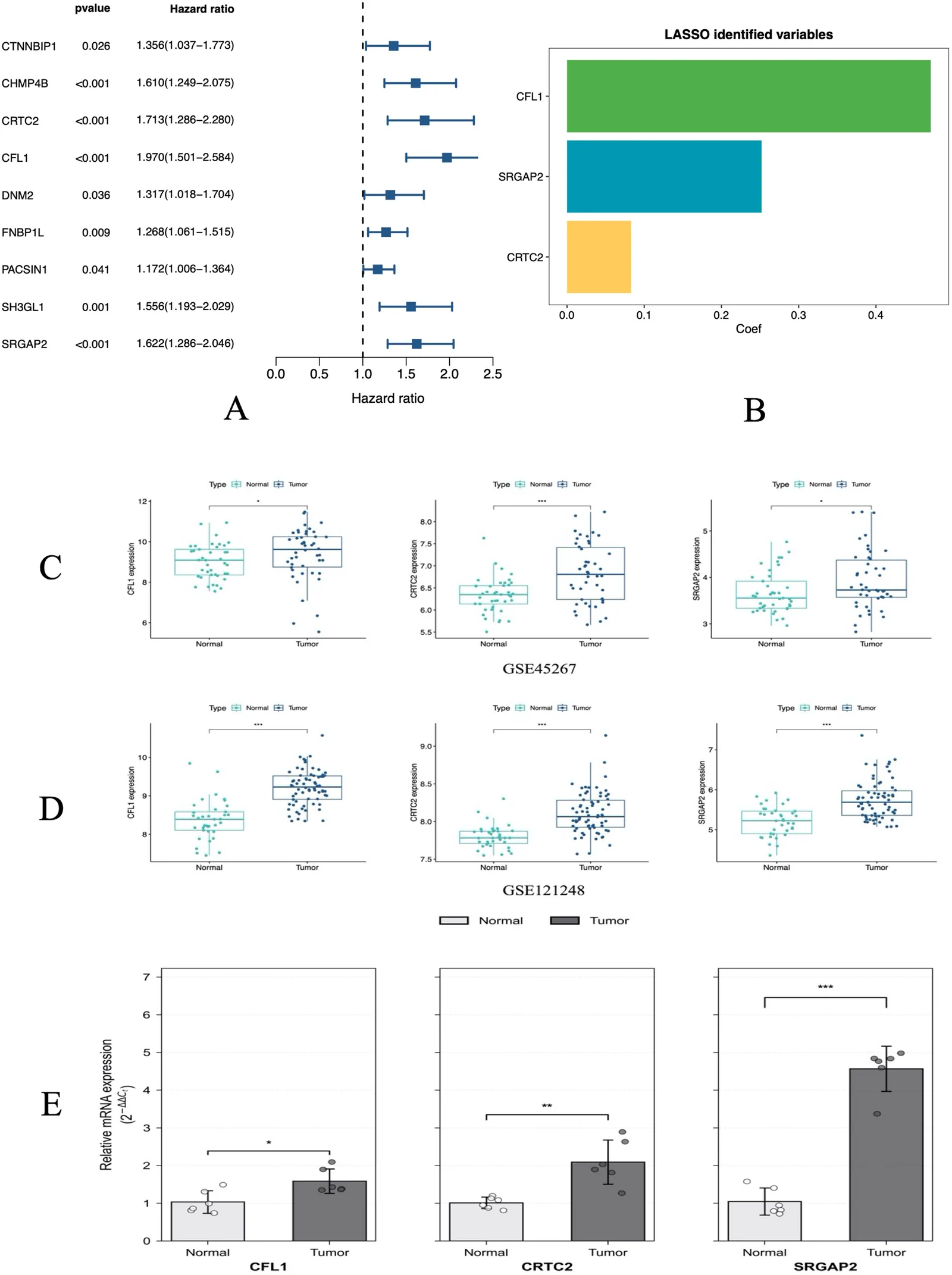
Identification and validation of the three-MTRG prognostic signature. **(A)** Forest plot of univariate Cox regression analysis for MTRGs associated with OS in TCGA-LIHC cohort. CFL1 showed the highest HR (1.970, P < 0.001). **(B)** LASSO-Cox regression selected three genes (CFL1, CRTC2, SRGAP2). Risk score = SRGAP2 × 0.252 + CRTC2 × 0.083 + CFL1 × 0.471. **(C, D)** Expression validation of the three genes in GSE45267 **(C)** and GSE121248 **(D)**. All genes were upregulated in tumor tissues. **(E)** qRT-PCR validation in 6 pairs of clinical HCC tissues. All three genes were overexpressed in tumors compared to normal controls. (*P < 0.05, **P < 0.01, ***P < 0.001).

### 3.2 Prognostic performance of the three-MTRG signature

Based on the median risk score, TCGA-LIHC patients were stratified into high- and low-risk groups. Principal component analysis (PCA) further confirmed effective separation between high- and low-risk groups in both TCGA and ICGC cohorts (**Supplementary Figure S1**). Kaplan-Meier analysis demonstrated significantly poorer OS in the high-risk group (P = 0.02, **Figure 3A**), with 5-year survival rates of 40% versus 10%, respectively. This prognostic separation was independently validated in the ICGC-LIHC cohort (P = 0.0052, **Figure 3B**). Time-dependent ROC analysis yielded 1-, 3-, and 5-year AUC values of 0.717, 0.649, and 0.635 in the TCGA cohort. As the number of patients with follow-up duration ≥5 years was insufficient in the ICGC-LIHC validation cohort, 1-, 3-, and 4-year AUC values of 0.706, 0.603, and 0.609 were calculated instead. The 1-year AUC exceeded 0.70 in both cohorts, indicating good predictive accuracy, particularly for short-term prognosis (**Figures 3C, D**). Risk score distribution and expression heatmaps confirmed that higher scores correlated with increased mortality and elevated expression of all three model genes in both cohorts (**Figures 3E, F**). Collectively, these findings establish the three-MTRG signature as a prognostic stratification tool with consistent performance across cohorts for HCC.

**Figure 3:**
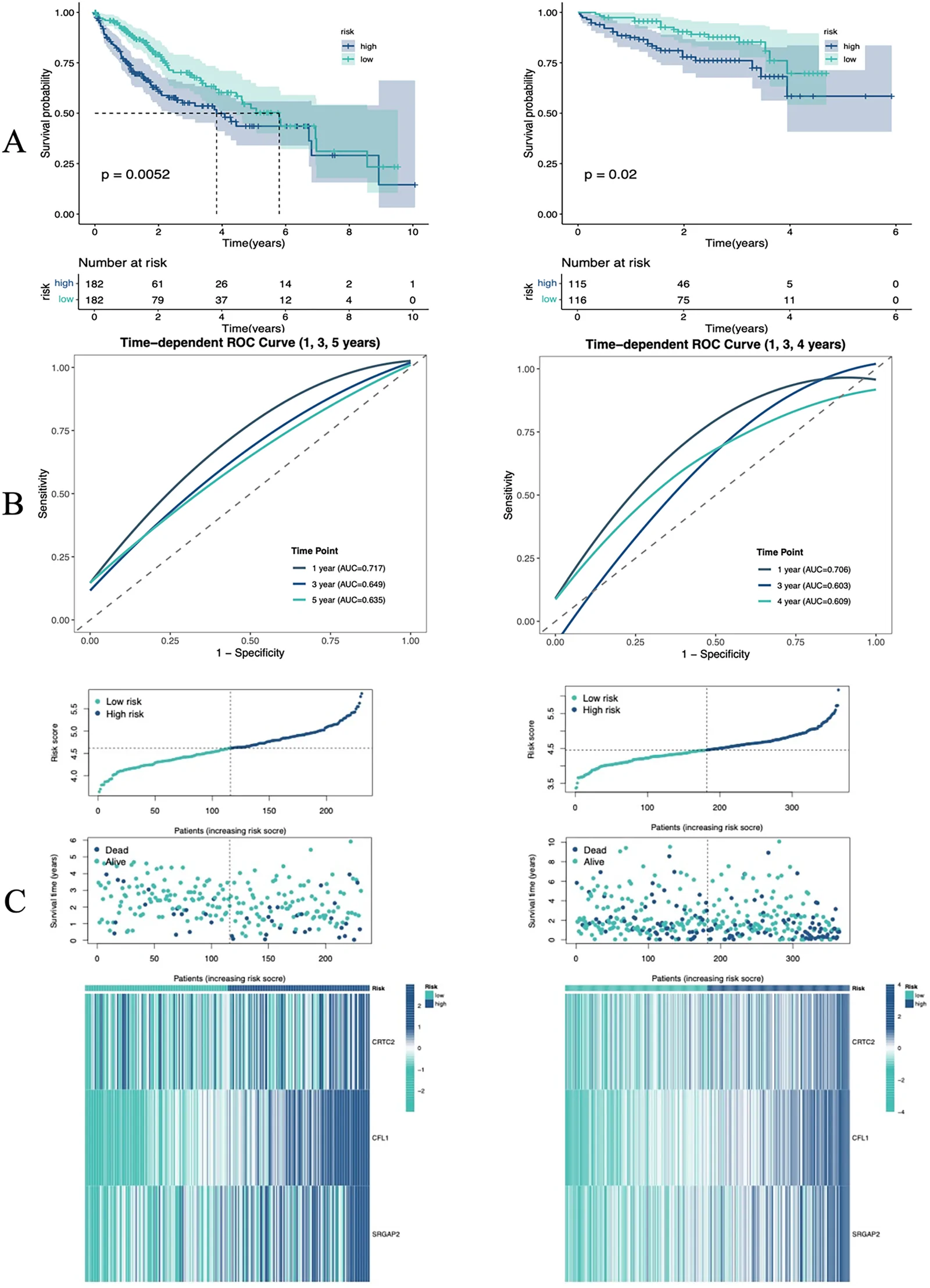
Prognostic performance of the three-MTRG signature in training and validation cohorts. **(A)** Kaplan-Meier survival curves for OS in TCGA-LIHC training cohort. High-risk group showed significantly poorer survival (P = 0.02); Kaplan-Meier survival curves for OS in ICGC-LIHC validation cohort. High-risk group showed significantly poorer survival (P = 0.0052). **(B)** Time-dependent ROC curves for 1-, 3-, and 5-year OS in TCGA cohort (AUC = 0.717, 0.649, and 0.635, respectively); Time-dependent ROC curves for 1-, 3-, and 4-year OS in ICGC cohort (AUC = 0.706, 0.603, and 0.609, respectively). **(C)** Distribution of risk scores, patient survival status, and expression heatmap of CFL1, CRTC2, and SRGAP2 in the TCGA cohort. Patients were sorted by increasing risk score; Distribution of risk scores, patient survival status, and expression heatmap of the three prognostic genes in the ICGC cohort. Higher risk scores were associated with increased mortality and elevated expression of all three model genes.

### 3.3 Construction and validation of a nomogram for individualized prognosis prediction

A nomogram integrating the MTRG risk score with clinicopathological features (age, gender, tumor stage, T stage) was constructed to facilitate individualized survival prediction (**Figure 4A**). The risk score contributed the widest point range (0–100), underscoring its dominant prognostic contribution. Time-dependent ROC curves demonstrated good discriminatory ability for 1-, 3-, and 5-year OS (**Figure 4B**), and calibration curves showed excellent agreement between predicted and observed survival probabilities (**Figure 4C**). Decision curve analysis further confirmed that the nomogram provided superior net clinical benefit over the risk score alone or individual clinicopathological variables. Univariate and multivariate Cox regression analyses were subsequently performed to assess the independent prognostic value of the risk score. Univariate analysis identified risk score (HR = 1.541, 95% CI: 1.330–1.786, P < 0.001), tumor stage (HR = 1.619, 95% CI: 1.290–2.032, P < 0.001), and T stage (HR = 1.553, 95% CI: 1.239–1.948, P < 0.001) as significant predictors of OS (**Figure 4D**). Multivariate analysis confirmed that the risk score remained an independent prognostic factor (HR = 1.449, 95% CI: 1.221–1.721, P < 0.001) after adjusting for clinical covariates (**Figure 4E**). Collectively, these findings establish the MTRG risk score as a consistent independent prognostic biomarker for HCC.

**Figure 4:**
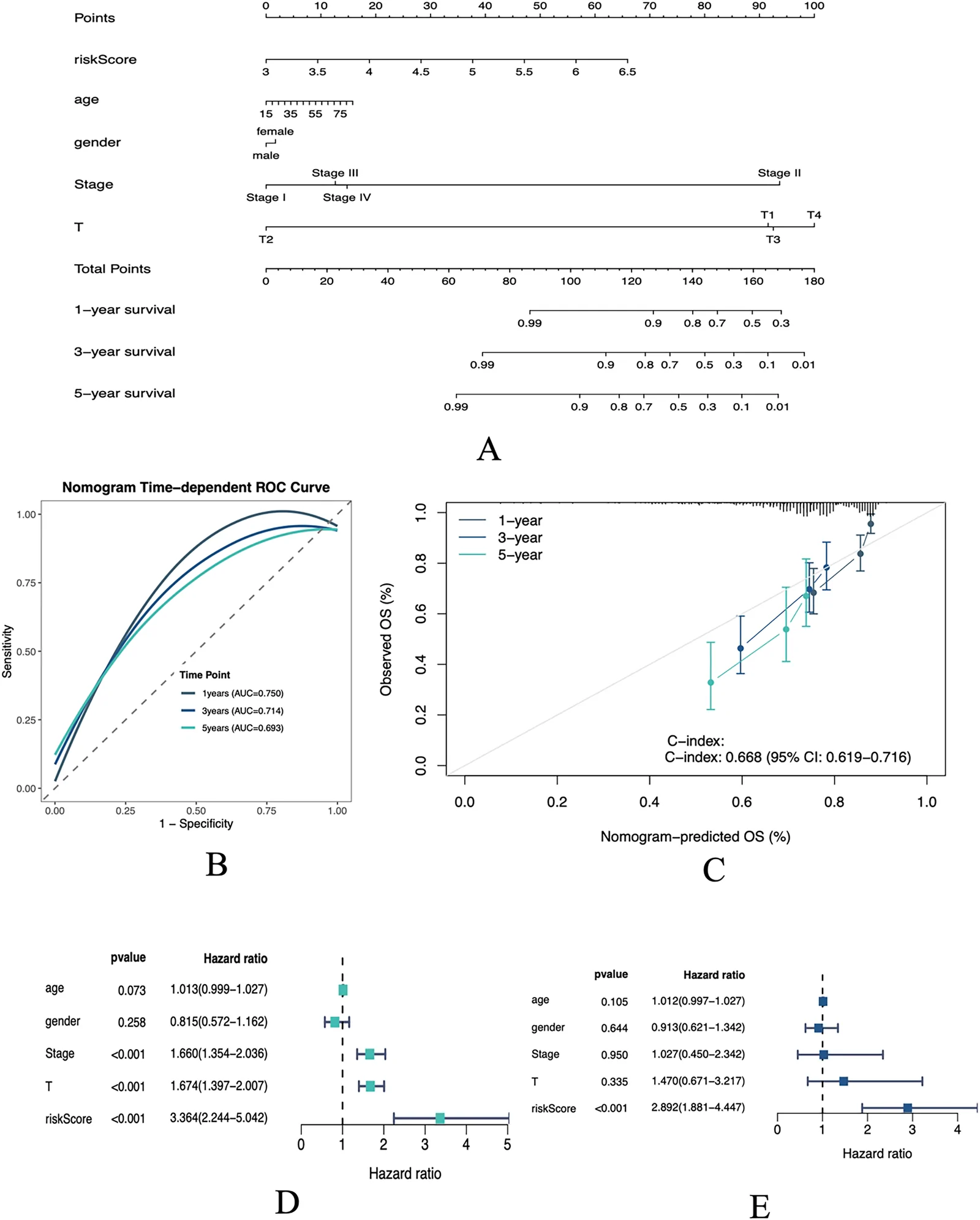
Nomogram for individualized survival prediction and its validation. **(A)** Nomogram integrating the MTRG risk score, age, gender, tumor stage, and T stage for predicting 1-, 3-, and 5-year OS in HCC patients. The risk score contributed the widest point range (0–100), indicating its dominant role in prognosis prediction. **(B)** Time-dependent ROC curves for the nomogram, demonstrating good discriminatory ability for 1-, 3-, and 5-year survival. **(C)** Calibration curves for the nomogram-predicted 1-, 3-, and 5-year OS. The predicted probabilities closely aligned with the ideal 45-degree reference line, indicating excellent calibration. **(D)** Univariate Cox regression forest plot showing that risk score, tumor stage, and T stage were significantly associated with OS. **(E)** Multivariate Cox regression confirming that the risk score remained an independent prognostic factor after adjusting for clinical covariates.

### 3.4 Immune landscape, immunotherapy response prediction, and GSEA enrichment

GSEA revealed that high-risk patients were significantly enriched in MTRG-related and ECM-related pathways, including ECM-receptor interaction (NES = 2.04, adjusted P = 7.09e-06), cytokine-cytokine receptor interaction (NES = 1.96, adjusted P = 8.10e-09), cell-adhesion molecules CAMs (NES = 1.70, adjusted P = 5.84e-04), focal-adhesion (NES = 1.57, adjusted P = 5.84e-04), and regulation of actin cytoskeleton (NES = 1.40, adjusted P = 1.68e-02) (**Figure 5A**), suggesting enhanced ECM remodeling and cytoskeletal reorganization in high-risk patients. Given CFL1’s dominant contribution to the risk score, we stratified patients by CFL1 expression to dissect the associated immune microenvironment. CIBERSORT analysis demonstrated that CFL1-high tumors exhibited significantly elevated Tregs and M0 macrophages, alongside reduced naive B cells, CD4 memory resting T cells, activated NK cells, and M1/M2 macrophages, while CD8^+^ T cell infiltration remained comparable between groups, indicating a Treg/M0-dominated immunosuppressive TME (**Figure 5C**). TIDE algorithm predicted significantly higher immune dysfunction/exclusion scores in high-risk patients (P = 9.8e-13, **Figure 5B**), with responder rates of 28% versus 57% in low-risk patients (**Figures 5D, E**), suggesting reduced likelihood of benefiting from ICB therapy. It is important to note that these TIDE-based predictions are computational and require validation in immunotherapy-treated cohorts. Additionally, combining TMB with risk score stratification further refined prognosis, with H-TMB/High-risk patients exhibiting the worst survival and L-TMB/Low-risk patients the best (P < 0.001, **Figure 4F**), offering potential value for outcome prediction.

**Figure 5:**
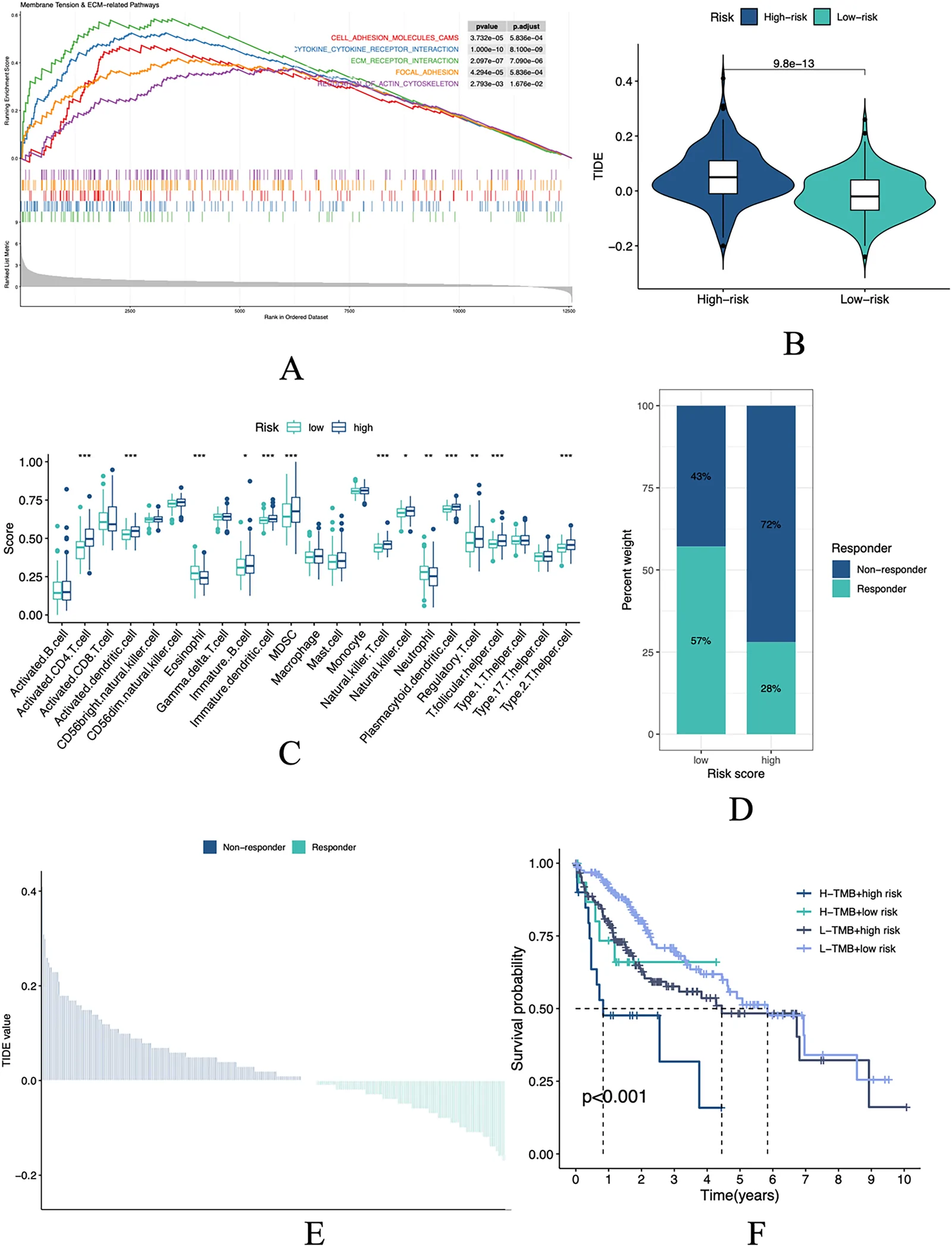
GSEA enrichment, immune landscape, immunotherapy response prediction, and TMB stratification. **(A)** GSEA enrichment plots for MT and ECM-related pathways, including ECM-receptor interaction, cytokine-cytokine receptor interaction, cell adhesion molecules CAMs, focal adhesion, and regulation of actin cytoskeleton. **(B)** Comparison of TIDE scores between high- and low-risk groups. High-risk patients had significantly higher TIDE scores (P = 9.8e-13). **(C)** Immune Cell Infiltration in CFL1-High versus CFL1-Low HCC Tumors. **(D)** Predicted immunotherapy response rates. Low-risk: 57% responders; High-risk: 28% responders. **(E)** Boxplot of TIDE scores between risk groups (P = 3.732e-05). **(F)** Kaplan-Meier survival curves for patients stratified by TMB and risk score. H-TMB + High-risk: worst prognosis; L-TMB + Low-risk: best prognosis (P < 0.001).

### 3.5 Single-cell transcriptomic landscape and CFL1-mediated cell-cell communication network in HCC

scRNA-seq analysis of the GSE242489 dataset identified 23,170 cells from normal liver and 18,076 from paired HCC tissues, which were clustered into major cell lineages including fibroblasts, endothelial cells, macrophages, T cells, NK cells, B cells, and hepatocytes (**Figures 6A, B**). Bulk-tissue Kaplan-Meier analysis confirmed that CFL1-high patients had significantly poorer OS (P < 0.001, **Figure 5C**), with marker gene validation supporting cluster annotation (**Figure 5D**). At single-cell resolution, UMAP plots revealed a distinct CFL1-high subpopulation separated from CFL1-low cells (**Figures 6E, F**). CellChat analysis demonstrated that CFL1-high cells exhibited more complex and intensive intercellular communication networks, with prominent outgoing signals toward fibroblasts, endothelial cells, macrophages, and T cells (**Figures 6G–J**). Notably, the MDK signaling pathway was identified as a key mediator, with significant upregulation of MDK-SDC2, MDK-NCL, and MDK-(ITGA4+ITGB1) ligand-receptor pairs in CFL1-high cells (**Figure 6K**), suggesting a potential role for CFL1-high tumor cells in MDK-mediated crosstalk with stromal and immune cells to remodel the tumor microenvironment.

**Figure 6:**
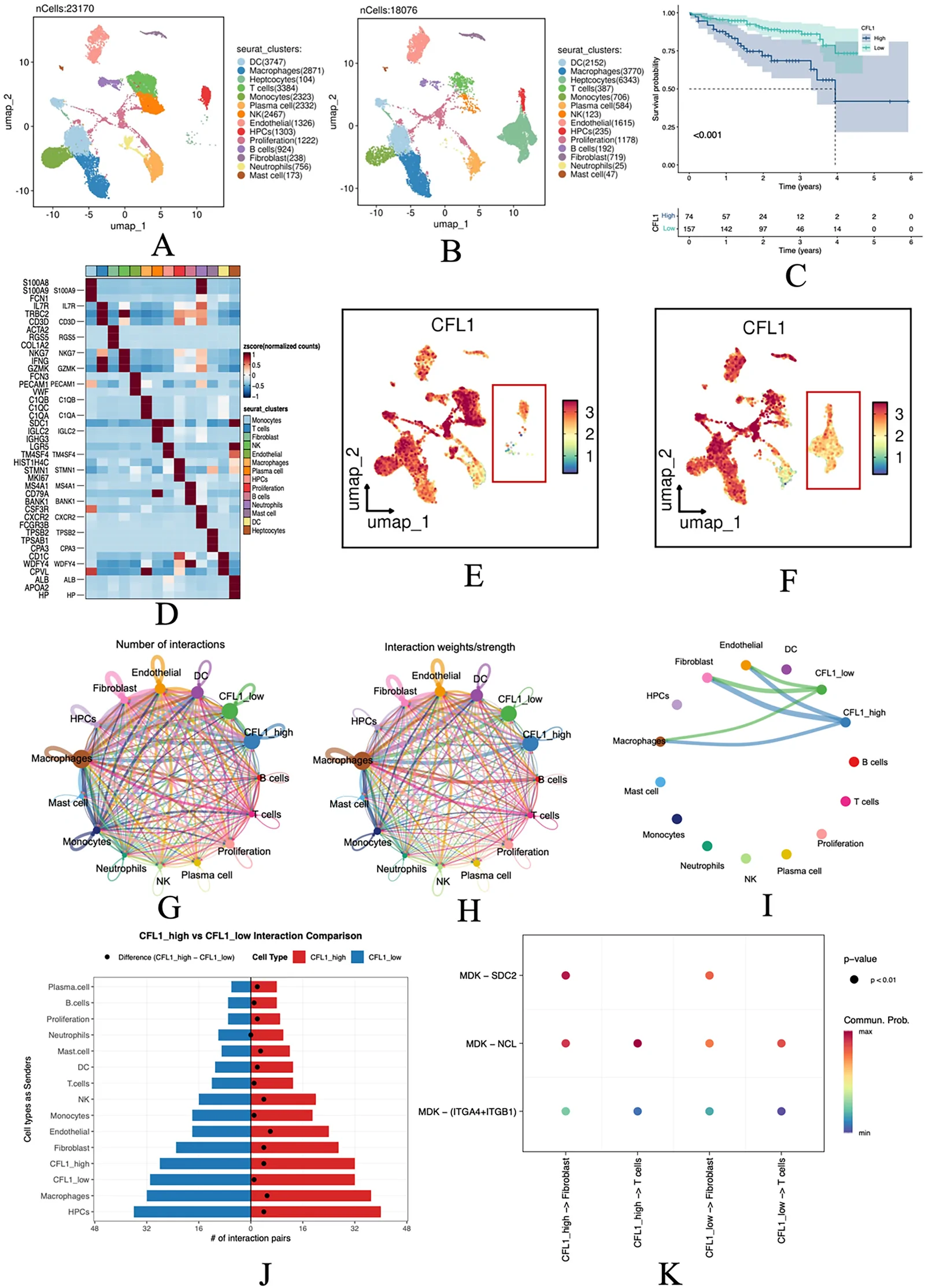
Single-cell transcriptomic landscape and CFL1-mediated cell-cell communication network in HCC. **(A)** UMAP plot of 23,170 cells from normal liver tissues, colored by cell type annotations. **(B)** UMAP plot of 18,076 cells from paired HCC tumor tissues, colored by cell type annotations. **(C)** Kaplan–Meier survival curves comparing OS between CFL1-high and CFL1-low patients in the TCGA-LIHC cohort (P < 0.001). **(D)** Marker gene heatmap validating the identity of each cell cluster. **(E)** UMAP plot showing CFL1 expression distribution across all cell types. **(F)** UMAP plot highlighting CFL1-high and CFL1-low cell subpopulations. **(G)** CellChat interaction network for CFL1-low group (number of interactions). **(H)** CellChat interaction network for CFL1-high group (number of interactions). **(I)** Simplified interaction network highlighting CFL1-high cells connecting to Fibroblasts, Endothelial cells, Macrophages, and T cells. **(J)** Bar plot comparing interaction counts between CFL1-high and CFL1-low groups across cell types. **(K)** Scatter plot of core MDK ligand-receptor pairs [MDK-SDC2, MDK-NCL, MDK-(ITGA4+ITGB1)], showing communication probability and significance between CFL1-high and CFL1-low groups.

### 3.6 Spatial transcriptomics reveals CFL1 enrichment in the stromal microenvironment

To investigate the spatial distribution of CFL1 within the HCC tumor microenvironment, we analyzed spatial transcriptomics data from the HCCDB database. Spots were annotated into four histopathological regions: Normal, Tumor, Stromal, and Immune. In the HCC-1L, HCC-1N, and HCC-1T samples, CFL1 exhibited the highest average expression in the Stromal regions (avg = 4.29, 4.02, and 4.16, respectively), with high spot purity (>93% in all samples), whereas expression in Tumor and Normal regions was considerably lower (**Figures 7A–F**). Similarly, in the HCC-5A, HCC-5B, HCC-5C, and HCC-5D samples, CFL1 expression was consistently elevated in Stromal regions (avg = 9.01, 7.01, 8.22, and 6.58, respectively) with high purity (84.62%, 67.06%, 73.48%, and 60.08%), while Tumor and Normal regions showed markedly lower expression levels (**Figures 7G, H**). These spatial transcriptomics findings demonstrate that CFL1 is predominantly enriched in the stromal compartment of the HCC microenvironment. It is important to note that while our single-cell CellChat analysis identified CFL1^+^ tumor cells as the primary source of MDK ligands, spatial transcriptomics revealed predominant CFL1 enrichment in stromal regions. We propose that this apparent discrepancy may reflect a CFL1-MDK-CFL1 paracrine feedback loop: CFL1^+^ tumor cells secrete MDK to activate adjacent fibroblasts, and MDK-stimulated fibroblasts in turn upregulate CFL1 expression, resulting in the observed stromal enrichment of CFL1. This interpretation is consistent with previous reports that mechanical stress can induce CFL1 expression in stromal cells (24). Notably, a paracrine feedback loop between tumor cells and fibroblasts has been demonstrated in gastric cancer, where tumor-derived activin B reprograms fibroblasts via NF-κB activation, and the activated fibroblasts in turn secrete IL-1β to reinforce tumor cell signaling, establishing a self-amplifying circuit that promotes tumor progression (25). This paradigm supports the plausibility of our proposed CFL1-MDK-CFL1 positive feedback loop, in which CFL1-high tumor cells may activate fibroblasts through MDK signaling, and activated fibroblasts in turn upregulate CFL1, thereby amplifying mechanical cues and ECM remodeling in the tumor microenvironment. However, this proposed feedback mechanism requires experimental confirmation through fibroblast-tumor co-culture and CFL1 overexpression/knockdown studies. This reciprocal crosstalk may amplify MT - driven ECM remodeling and contribute to tumor progression.

**Figure 7:**
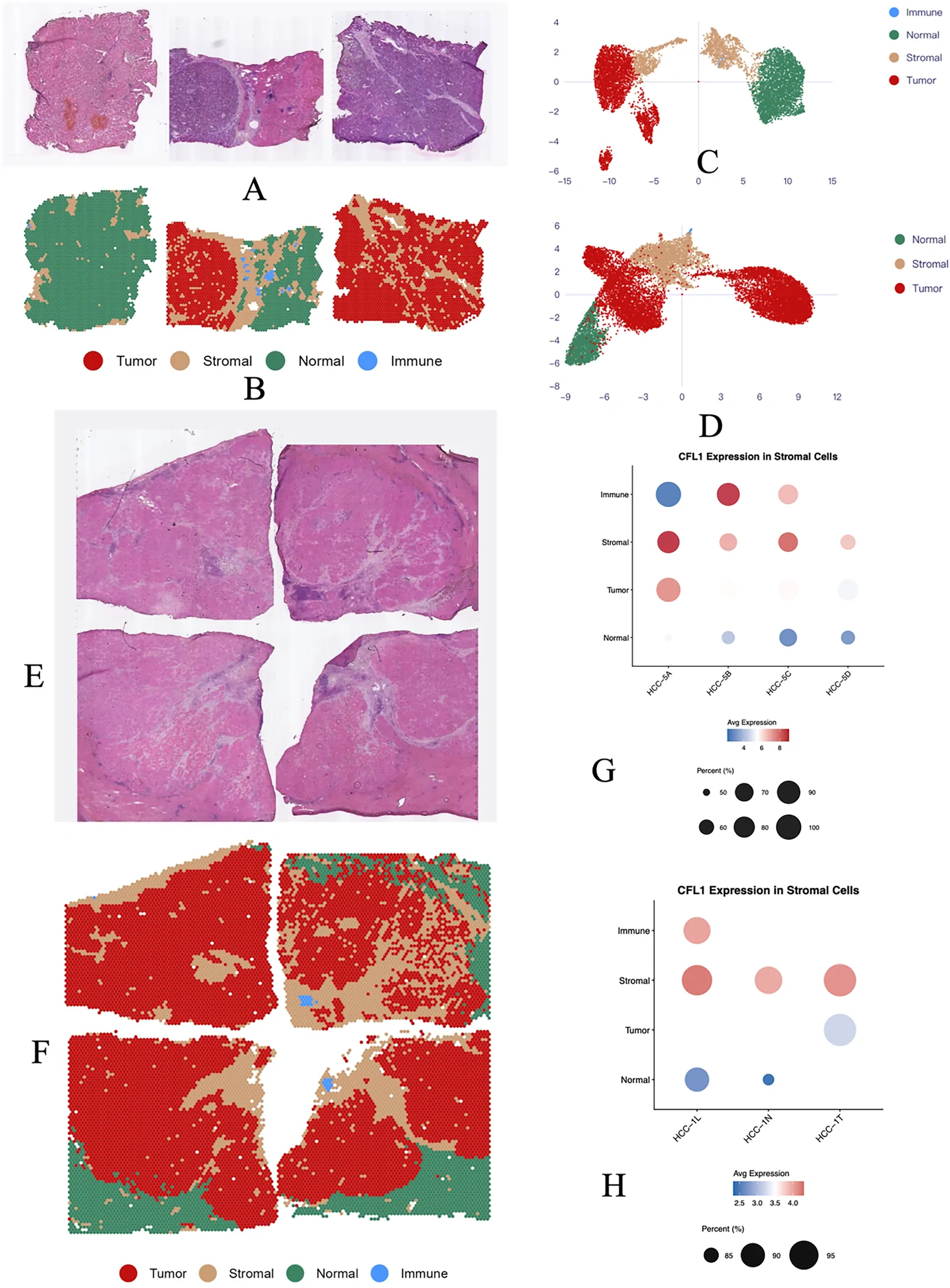
Spatial transcriptomics reveals predominant CFL1 enrichment in the stromal microenvironment. **(A–F)** Spatial CFL1 expression patterns across histopathological regions (tumor, stroma, normal, immune) in HCC-1L, HCC-1N, and HCC-1T, respectively. CFL1 exhibited the highest expression in stromal regions (avg = 4.29, 4.02, and 4.16), with spot purity >93%;Spatial CFL1 expression patterns in HCC-5A, HCC-5B, HCC-5C, and HCC-5D, respectively. CFL1 was consistently elevated in stromal regions (avg = 9.01, 7.01, 8.22, and 6.58), with purity ranging from 60.08% to 84.62%. **(G-H)** Quantification of average CFL1 expression across tumor, stromal, and normal regions in all seven samples.

## 4 Discussion

We established a three-MTRG prognostic signature that stratifies HCC patients and is associated with predicted immunotherapy resistance by TIDE analysis. By integrating single-cell and spatial transcriptomics, we further identified a candidate CFL1--MDK--stroma axis based on computational analyses through which MTRG expression shapes tumor-stroma crosstalk and immune evasion. The prognostic signature performed robustly across independent cohorts, with CFL1 emerging as the dominant contributor (weight = 0.471, HR = 1.970). This aligns with the established role of CFL1 as an actin-binding protein that regulates cytoskeletal dynamics and mechano-transduction. The nomogram integrating this signature with clinical variables provides a practical tool for individualized risk stratification, and decision curve analysis supports its clinical utility beyond conventional staging systems. Mechanistically, our single-cell analyses revealed that CFL1-high tumor cells engage in intensive intercellular communication via MDK signaling, targeting fibroblasts through MDK–SDC2, MDK–NCL, and MDK–(ITGA4+ITGB1), and T cells through MDK–NCL. MDK is a heparin-binding growth factor known to promote proliferation, migration, and angiogenesis (26–34), but its connection to MT signaling in HCC has not been previously reported. Notably, NF-κB signaling is a well-established oncogenic pathway in HCC. WDR54 was recently shown to promote HCC progression by enhancing NF-κB signaling (35), supporting a potential link between MDK signaling and NF-κB activation in our proposed CFL1–MDK–stroma axis. The spatial enrichment of CFL1 in stromal regions—despite its transcriptional origin in malignant cells—appears paradoxical but may be explained by a paracrine feedback loop: CFL1-high tumor cells may secrete MDK to activate cancer-associated fibroblasts, which in turn upregulate CFL1 and remodel the extracellular matrix. This reciprocal crosstalk could potentially amplify mechanical cues within the TME, consistent with our GSEA findings of enriched ECM–receptor interaction and focal adhesion pathways in high-risk patients. Such a mechanism also aligns with the known induction of CFL1 in stromal cells by mechanical stress (36) and provides a biophysical basis for tumor-stroma co-evolution in HCC. From an immunological perspective, CFL1-high tumors exhibited an immunosuppressive landscape dominated by Tregs and M0 macrophages, with comparable CD8+ T-cell infiltration but significantly higher TIDE scores indicative of functional exhaustion or exclusion. These findings are consistent with recent reports that matrix stiffness and MT can directly impair T-cell signaling and promote immune evasion (37, 38). The TIDE-predicted response rate to immune checkpoint blockade (28% versus 57%) in high-risk patients may suggest that the CFL1–MDK–stroma axis may constitute a mechano-immunological barrier to effective immunotherapy.

This study has several limitations. First, the proposed CFL1–MDK–stroma feedback loop and its immunomodulatory consequences require experimental validation in co-culture systems and orthotopic models. Second, the spatial transcriptomic analysis was based on a limited sample set; larger cohorts are needed to confirm the observed spatial distribution. Third, the precise molecular mechanism by which CFL1 regulates MDK secretion, and how MDK signaling reciprocally affects CFL1 in fibroblasts, remains to be determined. Fourth, the proposed CFL1-MDK-stroma positive feedback loop is based entirely on in silico analyses and remains a computational hypothesis. Experimental validation through tumor-fibroblast co-culture assays, CFL1 overexpression/knockdown experiments to detect MDK secretion, and assays measuring CFL1 changes in fibroblasts upon MDK stimulation are required to confirm this mechanism. Despite these limitations, our findings have direct translational relevance. The three-MTRG signature and nomogram offer clinically accessible tools for prognosis prediction and treatment guidance. From a clinical translational perspective, this MTRG-associated signature may offer several potential applications. First, it could be used to stratify HCC patients into distinct prognostic subgroups, potentially identifying high-risk patients who may benefit from more aggressive postoperative surveillance and adjuvant therapy. Second, the association between high-risk scores and predicted immunotherapy resistance suggests that this signature may help identify patients who are less likely to benefit from immune checkpoint blockade monotherapy and may instead require combination therapeutic strategies. Third, the identification of the CFL1-MDK-stroma axis suggests that CFL1-high patients might represent a candidate subgroup for mechano-modulatory therapy combined with immune checkpoint blockade, although this requires prospective validation. However, several practical limitations must be acknowledged. The signature relies on transcriptome profiling, which requires RNA sequencing infrastructure that may not be universally available in clinical settings. Development of a simplified assay targeting the three-gene panel (e.g., qRT-PCR or targeted RNA panel) would facilitate clinical translation. Additionally, the model has not been validated in prospective cohorts, and its performance in real-world clinical settings requires further evaluation. The identification of the CFL1--MDK--stroma axis suggests a potential therapeutic direction: pharmacological targeting of CFL1 or MDK signaling may simultaneously disrupt tumor-stroma crosstalk and alleviate immune suppression. However, this remains a hypothesis that requires preclinical validation. Given that MDK inhibitors have shown preclinical efficacy in other malignancies (33, 39, 40), CFL1-high HCC patients could potentially represent a responsive subgroup for combination therapy warranting further investigation that pairs mechano-modulatory agents with immune checkpoint blockade.

## 5 Conclusion

This study establishes an MTRG-associated prognostic signature and suggests a potential CFL1--MDK--stroma axis that may bridge biophysical cues with tumor-stroma co-evolution and predicted immunotherapy resistance by computational analysis in HCC. Functional validation and preclinical evaluation of targeted interventions against this axis are now warranted.

## Data Availability

The original contributions presented in the study are included in the article/**Supplementary Material**. Further inquiries can be directed to the corresponding authors.
