# Supplementary material for: Integrative multi-omics analysis reveals a membrane tension signature associated with tumor-stroma crosstalk in hepatocellular carcinoma
Source: Front Immunol. 2026 Oct 5;17:1978174. doi: 10.3389/fimmu.2026.1978174 (PMC13677693; doi:10.3389/fimmu.2026.1978174)
Supplement: Supplementary file 1 [file Table1.docx]

| RHOA | FLNA | STK | PSTPIP1 |
| --- | --- | --- | --- |
| CTNNBIP1 | MYO1C | FNBP1 | PSTPIP2 |
| EZR | EPO | MTSS1L | SH3GL1 |
| RDX | FLG | TRIP10 | SH3GL1P1 |
| MSN | MTSS2 | CRTC2 | SH3GL1P2 |
| MYO1A | PDCD6IP | CFL1 | SH3GL1P3 |
| GSK3b | PICK1 | CAPZB | SH3GL2 |
| FGFR1 | STK10 | DIAPH1 | SH3GL3 |
| GBF1 | ACAP2 | MYH9 | SH3GLB1 |
| BAIAP2 | AMPH | MYH10 | SH3GLB2 |
| ARF1 | ARFIP1 | PIEZO1 | SNX1 |
| CDC42 | BIN1 | SDC4 | SNX2 |
| ACTR2 | FCHO1 | PECAM1 | SNX3 |
| ACTR3 | FCHO2 | CDH1 | SNX4 |
| PICK1 | FNBP1L | ITGB1 | SNX5 |
| EGF | ICA1 | FERMT2 | SNX6 |
| CHMP4B | NOSTRIN | TIMP1 | SNX9 |
| EGFR | PABPC4 | CAV1 | SRGAP1 |
| TSG101 | PACSIN1 | DNM2 | SRGAP2 |
| ALIX | PACSIN2 | MYO1B | SRGAP3 |
| SLK | PACSIN3 | ARHGAP26 | SPNPEP2 |

**Supplementary Table S1. List of 84 membrane tension-related genes (MTRGs).**

**Supplementary Figure S1. Principal component analysis (PCA) of high- and low-risk groups in the TCGA and ICGC cohorts.**

(A–B) PCA plots showing effective separation between high- and low-risk patients in the TCGA-LIHC cohort. (C–D) PCA plots showing effective separation between high- and low-risk patients in the ICGC-LIHC cohort.
